# Long-Term Randomized Controlled Trials of Diet Intervention Reports and Their Impact on Cancer: A Systematic Review

**DOI:** 10.3390/cancers16193296

**Published:** 2024-09-27

**Authors:** Edward R. Sauter, Gisela Butera, Tanya Agurs-Collins

**Affiliations:** 1 Division of Cancer Prevention, National Cancer Institute/National Institutes of Health (NIH), 9609 Medical Center Drive, Rockville, MD 20850, USA; 2 Office of Research Services, NIH Library, Bethesda, MD 20892, USA; gisela.butera@nih.gov; 3 Division of Cancer Control and Population Sciences, National Cancer Institute/National Institutes of Health (NIH), 9609 Medical Center Drive, Rockville, MD 20850, USA; collinsta@mail.nih.gov

**Keywords:** cancer risk reduction, low caloric diet, Mediterranean diet, low-fat diet, systematic review

## Abstract

**Simple Summary:**

Lifestyle dietary changes implemented by people who are overweight or obese to improve health generally focus on caloric restriction, changes in macronutrient intake, or both. Findings from reports of the impact of these changes on cancer are mixed, with many subjected to secondary analysis bias. An impact on cancer endpoints requires long-term (≥1 year) intervention. There is a clear need for more reports evaluating the role of diet in cancer risk reduction in overweight/obese individuals who are at increased risk of obesity-related cancers, especially reports focusing on cancers other than breast. Pharmacologic and metabolic/bariatric surgery-MBS strategies have demonstrated promise to deliver both greater initial weight loss and better weight loss maintenance). The impact of weight loss on reducing cancer risk may depend on the (1) amount and (2) maintenance of weight loss.

**Abstract:**

Background: Most randomized controlled trials (RCTs) assessing the impact of diet on cancer have been short term (<1 year), mostly evaluating breast cancer survivors. Given the many-year interval that is generally required for an intervention to have an impact on cancer risk or prognosis, as well as the fact that lifestyle strategies such as diet modification frequently fail due to lack of adherence over the long term, we focused this systematic review only on longer-term (≥1 year) intervention reports. Diet intervention reports focused on reducing cancer risk in overweight and obese individuals target caloric restriction (every day, some days, or most hours of each day). Methods: This study is a systematic review of RCTs lasting at least 1 year, testing dietary interventions with a primary or secondary endpoint of cancer or a biomarker linked to cancer. Results: Fifty-one reports met our review criteria. Twenty of fifty-one (39%) reports are RCTs where the primary endpoint was cancer or a cancer-related biomarker, while the other reports evaluated reports where cancer or a cancer-related biomarker was a secondary endpoint. Thirteen of twenty (65%) primary reports evaluated isocaloric, and the remaining eight evaluated low-calorie diets. All but one of the primary and two secondary isocaloric diet reports evaluated the benefit of a low-fat diet (LFD), with the other three evaluating a Mediterranean diet (MedD). More LCD vs. isocaloric diet primary reports (71% vs. 38%) demonstrated cancer or cancer-related biomarker benefit; the difference in chance of benefit with secondary reports was 85% for LCD vs. 73% for isocaloric diets. Three of three MedD reports demonstrated benefit. Sixty-nine percent (20/29) of the secondary reports came from two large reports: the WHI diet modification trial (15 secondary reports) and the polyp prevention trial (5 secondary reports). Nineteen of twenty-two (86%) primary reports enrolled only women, and three enrolled both men and women. No study that met our criteria enrolled only men, comprising 1447 men in total vs. 62,054 women. Fifteen of twenty (75%) primary reports focus on healthy women or women with breast cancer. Adherence findings are discussed when provided. Conclusions: More long-term RCTs evaluating cancer and cancer-related biomarker endpoints are needed, especially for cancers at sites other than the breast.

## 1. Introduction

According to the American Cancer Society (ACS), no less than 18% of all cancers and about 16% of U.S. cancer deaths are linked to overweight, physical inactivity, alcohol consumption, and/or poor nutrition [1]. Diet recommendations to mitigate cancer risk have been published by multiple societies, including the ACS and the American Institute for Cancer Research (AICR). The ACS recommendations include maintaining a healthy weight and avoiding weight gain, being physically active, and choosing a diet that focuses on healthy foods (including a variety of vegetables, legumes, fruits, and whole grains) for cancer prevention and control [1]. AICR recommendations mostly mirror those of the ACS, though the AICR recommendations specifically address the breast cancer-reducing benefits of breastfeeding https://www.aicr.org/cancer-prevention/ (accessed on 5 April 2024). Thus, public health recommendations encourage the adoption of healthy dietary patterns and maintaining a healthy weight for cancer risk reduction. High vs. low adherence to diet and cancer risk reduction guidelines have consistently been associated with significant reductions in overall as well as endometrial, breast, and colorectal cancer risk and survival [2].

Many diets have been proposed for caloric restriction (CR) and/or a healthy lifestyle by altering macronutrient intake, though there is no consensus regarding whether one diet is better than another regarding weight loss or cancer risk reduction and control. “There have been no rigorous, long-term reports comparing contenders for best diet laurels using methodology that precludes bias and confounding, and for many reasons such reports are unlikely” [3]. Moreover, dietary adherence assessment is not consistently measured or consistently reported in published studies, hindering the ability to compare adherence between dietary approaches. Commonly used diets target weight loss and/or metabolic dysfunction since obesity and metabolic dysfunction are known to impact multiple common diseases such as cancer, diabetes mellitus, and cardiovascular disease (CVD) [4]. We conducted a systematic review of the literature to examine the effect of long-term (≥1 year) dietary intervention reports on cancer and cancer-related biomarkers.

## 2. Weight Loss and Altered Macronutrient Diets

Below we briefly address low-calorie and isocaloric diets, followed by the published studies that we identified in our systematic review. In our review of each study, we were disappointed to find the reporting of diet adherence was inconsistent from study to study, making comparisons between dietary approaches difficult. As it turns out, this lack of consistency is a recognized problem worldwide, and there has been a call by the Food and Agriculture Organization of the United Nations and the World Health Organization to provide more uniform dietary metrics [5].

### 2.1. Low Calorie Diet (LCD)

LCDs focus on weight loss. Caloric restriction is an important strategy to reduce overweight and obesity-associated metabolic and inflammatory perturbations associated with cancer risk [6]. LCDs focus on the restriction of calories each day, for some days per week or month (intermittent fasting [IF]), or for most hours each day (time-restricted eating, a form of IF). Diets that alter macronutrients for health benefits may also include caloric restriction, whether or not intentional. For those who have overweight or obesity, the goal is often to lose weight, resulting in a metabolically beneficial effect.

### 2.2. Low Fat Isocaloric Diet

Low-fat diets (LFDs) have been promoted for a healthy heart and metabolic improvement, which impacts diabetes and cancer risk. One of the largest of these reports focusing on cancer was the Women’s Health Initiative diet modification trial, which enrolled and randomized healthy postmenopausal women with a fat intake at baseline of at least 32% of their daily calories to a usual diet (control) or intervention (fat intake 20% of energy, with increased vegetables, fruits, and grains). Although the study was not designed to achieve weight loss, the investigators found a mean 3% (2.2 kg) lower body weight after one year in the intervention group (*p* < 0.001) [7]. After 8.5 years of study, breast cancer incidence and deaths were nonsignificantly lower in the intervention group. After 19.6 years of median follow-up, the reduction in deaths from breast cancer in the intervention group was significant (*p* = 0.02) [7].

### 2.3. Mediterranean (MedD) Isocaloric Diet

Started in 1947, the Seven Countries Study (SCS) found that a diet including olive oil, whole grains, fruits, vegetables, seafood, beans, and nuts that were widely consumed in Greece, southern Italy and Crete (MedD) resulted in the lowest rates of CVD and longest life expectancy [8]. Since the SCS, multiple reports have correlated increased adherence to a MedD with lower rates of CVD [8] and cancer [9], including colorectal (HR = 0.82), breast (HR = 0.92), gastric (HR = 0.72), and liver cancer (HR = 0.58).

The PREDIMED study was conducted to identify participant characteristics and study features that predict short and long-term adherence to a MedD [10] (Table 1). After 12 months of study, improved adherence to a MedD was greatest among men who were nondiabetic and among those with worse baseline dietary habits, whereas among women, improved compliance was greatest among those who were married and those with worse baseline dietary habits [11].

## 3. What Predicts Long-Term Dietary Adherence?

This report focuses on reports lasting at least 1 year since dietary interventions take years to impact cancer risk. There are multiple impediments to dietary adherence, many of which are psychological, including depression, emotional eating, and poor exercise attitude and adherence [62]. Short-term success in diet adherence has been associated with openness to change, setting ambitious targets, and rewarding weight loss, but often, there is weight regain [63].

Many of the challenges with long-term adherence to lifestyle changes are similar to those related to short-term adherence, including challenges associated with adherence to lifestyle behaviors recommended for weight loss and healthy weight management (i.e., reductions in dietary intake and increases in physical activity). Given the limited number of reports evaluating long-term diet adherence, a question arises regarding whether short-term adherence provides longer-term benefits. Although the primary outcome was not a cancer endpoint, the findings of the POUNDS LOST study provide insight into the question of short- vs. long-term adherence. The study correlated diet self-monitoring and adherence during the first 6 months with changes in adiposity and CV risk factors at 24 months [64]. Early adherence was associated with changes in weight loss and waist circumference at 6 and 24 months but not with adiposity or with CV risk factors, suggesting that short-term adherence provides some, but perhaps not an optimal, long-term benefit. The Look AHEAD trial found that individuals with the greatest weight loss during the first 2 months of the intervention were more likely to achieve ≥5% weight loss through year 8, and weight loss < 3% at 2 months was associated with poor adherence to intervention meetings, fewer meal replacements, and less physical activity than those with higher initial weight loss [65]. Thus, early adherence was associated with longer-term weight loss maintenance in this study.

## 4. Systematic Review Methodology

The aim of this systematic review is to investigate how long-term diet reports are related to cancer risk and control. The review was performed according to the Preferred Reporting Items for Systematic Reviews and Meta-Analyses (PRISMA) 2020 and the protocol registered in PROSPERO CRD42023438966 (PRISMA checklist available in Supplementary File S1). We selected manuscripts for review based on the criteria outlined in Supplementary Table S1, with results listed in Table 1. Briefly, we included only randomized clinical trials (RCTs) that enrolled adults in which the study evaluated the impact of a long-term dietary intervention (>1 year) on cancer or cancer-related biomarkers.

The search strategy was developed by two authors (ERS and TAC) in collaboration with a professional research librarian with expertise in systematic reviews (GB) using an iterative process and further peer reviewed. A combination of indexed terms (e.g., MeSH) and keywords were used to create a PubMed search strategy that was translated into other databases using appropriate syntax and controlled vocabulary. The following biomedical databases were searched: MEDLINE via PubMed, EMBASE (Elsevier), and Web of Science (Clarivate). The search was limited to English and human reports with no date restrictions. In addition, we performed a citation search of the references of included articles. The complete database search strategies can be found in Supplementary File S1.

**Figure 1:**
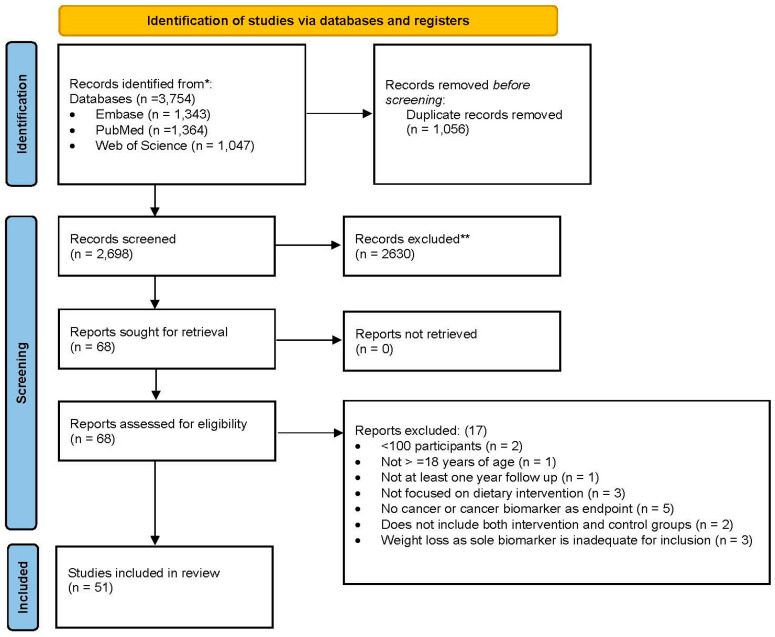
PRISMA 2020 flow diagram for new systematic reviews which included searches of databases and registers only. From [66].

For the study selection process, duplicates were removed using EndNote 21, and records were then imported into Covidence screening software (Web based version of Covidence 2024, Melbourne, Victoria, Australia: https://www.covidence.org) for the screening of records. Two authors (ERS and TAC) independently screened titles and abstracts as well as full-text records according to eligibility criteria. Any disagreements were resolved through consensus among the authors. The screening process for included articles is available in the PRISMA flow diagram (Figure 1). Data extraction from the included articles was performed by two authors (ERS and TAC) and extracted to a predefined spreadsheet.

The risk of bias (RoB) assessment was carried out using the NHLBI Study Quality Assessment Tools to assess the quality of RCTs) [9]. The RoB assessment was used to assess the overall quality of the evidence of the included reports and investigate whether potential heterogeneity could be explained by a difference in study risk of bias. Two reviewers (ERS and TAC) independently performed RoB and met to compare and resolve disagreements through discussion (available in Supplementary File S1).

## 5. Results

Given the time gap between diet intervention initiation and a measurable impact on cancer risk, many short-term reports comparing the benefits of various diets focused on non-cancer endpoints and were not included in the systematic review. The focus of our systematic review was on RCTs (i.e., reports with individuals randomized to at least two arms) lasting at least 1 year. We identified a limited number of RCTs; a total of 51 articles [7,12,13,14,15,16,17,18,19,20,21,22,23,24,25,26,27,28,29,30,31,32,33,34,35,36,37,38,39,40,41,42,43,44,45,46,47,48,49,50,51,52,53,54,55,56,57,58,59,60,61] were included that evaluated the impact of long-term lifestyle dietary changes on cancer or cancer-related biomarkers (Table 1). We also included RCTs with a non-cancer primary endpoint where participants were followed over time to assess cancer or cancer-related biomarkers as secondary endpoints to gain a better understanding of how studies involving lifestyle dietary changes impacted cancer or cancer-related biomarkers. Statistical analysis attempted to address bias linked to prior knowledge of the data, which is an important approach to minimize results bias [67].

Thirteen of twenty (65%) primary reports evaluated isocaloric and the remaining seven LCDs (Table 2).

All but one primary and two secondary isocaloric diet reports evaluated the benefit of an LFD, with the other three evaluating an MedD. Whereas more LCD primary reports vs. isocaloric diets (71% vs. 38%) demonstrated a cancer or cancer-related biomarker benefit, less difference in benefit was observed in secondary reports between LCD and isocaloric diets (86% vs. 73%, respectively). All three MedD reports demonstrated benefit. Sixty-nine percent (20/29) of the secondary reports came from two large reports: the WHI diet modification trial (15 secondary reports) and the polyp prevention trial (5 secondary reports). Sixteen of twenty (80%) studies focused on either healthy women or women with breast cancer (Table 3).

Of the primary reports, 80% enrolled only women and 20% both men and women, while no study that met our criteria enrolled only men. Primary reports in our review enrolled a total of 1447 men vs. 62,054 women.

## 6. Discussion

The findings from our systematic review highlight a number of points. First, most long-term RCTs, both primary and secondary reports, that met our criteria focused on healthy women or women with breast cancer. Because the reports overwhelmingly focus on women at risk for or with breast cancer, we have little to no RCT long-term trial information among men on the role of lifestyle changes on cancer, either risk or prognosis. This is especially important because men are more likely than women to develop cancer. Specifically, in the U.S., the rate of new cancer cases per 100,000 persons was 478.7 for men vs. 416.7 for women for the years 2017–2021 [68]. Moreover, the death rate from cancer is higher among U.S. men than women (173.2 vs. 126.4 per 100,000) [69]. Comparing sexes based on race/ethnicity, mortality from cancer was highest among non-Hispanic black men (208.3 per 100,000) and lowest among Asian/Pacific Islander women (82.6 per 100,000) [69]. Despite this, none of the primary reports that met our criteria focused on men, and the total number of men enrolled in primary reports in our review was only 2% vs. 98% for women. This disparity has been noted by many in the scientific community. Investigators are now addressing possible mechanism(s) for sex differences in cancer [70], risk of severe adverse events after treatment for cancer [71], outcomes in oncology clinical trials [72], and the difference in cancer incidence after receipt of an organ transplant [73]. This disparity has been noted by the National Institutes of Health (NIH). The NIH Sex and Gender Differences in Cancer Workshop Series was conducted in February through June 2024. There is also a trans-National Cancer Institute extramural awareness group that hosts webinars on sex differences in cancer.

For primary reports, it appeared that LCDs were more likely to have a beneficial impact on cancer risk vs. cancer biomarkers, whereas the difference was far less pronounced for reports where cancer was not the primary outcome of the study. This is not surprising, given their large size and long-term follow-up. Secondary data analyses, while providing the potential to answer important questions, have a risk of bias that likely exceeds the analysis of the primary outcome stated in an RCT [67]. This includes researcher bias, including analyzing data likely to demonstrate significant results, the tendency to focus on evidence that is consistent with one’s beliefs (confirmation bias), selective reporting only when the findings are significant. As a result, secondary data analysis results are often not able to be replicated [67]. Given this, it is our view that the secondary findings comparing the efficacy of LCD vs. isocaloric diets should be viewed with a level of caution, likely requiring confirmation before being viewed as true.

The identification of cancer risk reduction from weight loss is generally related to both the maximum amount of weight loss and the ability to maintain that weight loss. Dietary strategies have strived to obtain and sustain 7% to 10% total body weight loss. Lifestyle reports suggest that at least 5% sustained weight loss is required to detect a reduction in the risk of cancer or cancer-related biomarkers [74]. Our systemic analysis indicates that diet intervention reports (>1 year) may achieve a 5% weight loss, but rarely 10% or more. This raises the question as to the type of strategies needed to increase long-term weight loss and potentially greater cancer risk reduction.

There is convincing evidence that metabolic/bariatric surgery (MBS), which generally results in greater and more sustained weight loss than nonsurgical weight loss strategies, leads to a significant reduction in obesity-related cancers [75]. Ten years after surgery, diabetes remission was associated with a 60% reduction in cancer risk [76]. The prolonged weight reduction after MBS among the severely obese has also been shown to reduce death from cancer [76]. The reduction in death from cancer after a median follow-up of 20 years from surgery was 23% [77].

There are now pharmacologic weight loss strategies centered on GLP-1 receptor agonists, which can lead to a mean of 10% weight loss for at least 4 years [78] as long as the individual continues on the agent. Among the reasons to stop these medications are (1) cost and (2) unknown side effects with long-term use. Stopping the agent generally leads to weight regain. It remains unclear if these strategies will be as effective as surgery for cancer risk reduction. Reports in the future will require long-term follow-up.

A 2006 viewpoint from Pagano and Appelhans called for an end to the diet debates [79] to identify the ideal diet for disease prevention and weight loss. They justify their stance by reporting that “Numerous randomized trials comparing diets differing in macronutrient compositions … demonstrated differences in weight loss and metabolic risk factors that are small and inconsistent.… The only consistent finding among the trials is that adherence was most strongly associated with weight loss and improvement in disease-related outcomes”. Notably, the metabolic factors evaluated were generally weight loss and laboratory findings such as lipid profiles rather than cancer risk reduction endpoints. A counter view was proposed in 2013 that diets are not equivalent regarding metabolic outcomes, citing a meta-analysis comparing low-carbohydrate and low-fat diets, which demonstrated different changes in lipid profiles based on diet [80] and therefore cautioned against healthcare providers advising patients to choose whichever diet they are most likely to adhere to was premature until more is known about the safety and efficacy of different diets [81]. Reports comparing the benefits of various diets regarding cancer risk reduction endpoints are sorely needed. The reports should compare their results to what is currently the gold standard for adherence and most associated with cancer risk reduction: diet adherence after MBS.

Limitations of the study include the lack of RCTs with cancer or cancer as a primary endpoint, the lack of diversity in the RCTs that we identified, and the lack of studies that address the effects of the same dietary strategy among different ethnic age groups or health conditions. Below, we discuss possible strategies to mitigate these limitations.

## 7. Conclusions and Future Directions

Predictors of diet adherence include several factors, including female sex, older age, diabetic, depression, body weight, physical activity, nonsmoker, white ethnicity, higher socioeconomic status, and being married [10]. Sustained weight loss has been difficult to maintain for most, if not all, weight loss diets. Recent reports of GLP-1 agonists among obese individuals with or without T2DM lasting over 1 year demonstrate encouraging adherence rates, though the individuals have extensive support while on study and may not accurately reflect real-world findings. Social support programs appear to increase adherence to diets but may be impractical in the very long term due to cost. Few reports have evaluated long-term (>1 year) diet adherence. Among the reports that report adherence, biomarker changes may be useful in the assessment of adherence. For reports where the primary goal is a reduction in weight, weight loss may be a useful surrogate assessment for dietary adherence. On the other hand, for diets where the primary goal is metabolic improvement rather than weight loss, weight loss may not be an optimal surrogate adherence marker. Overweight and obese individuals who meet the criteria (BMI ≥ 27 with one or more weight-related comorbidities) may be directed toward pharmacologic therapy. Severely obese (a group likely to fail a long-term dietary strategy) individuals who meet the criteria (current criteria ≥ 35 BMI and considered for people with a BMI 30–34.9 with metabolic disease and fit for surgery; https://asmbs.org/news_releases/after-30-years-new-guidelines-for-weight-loss-surgery (accessed on 9 May 2024) could be encouraged to consider MBS.

Currently, findings from diet intervention reports have generally not assessed cancer risk reduction as their primary outcome because the effects of obesity on cancer risk take longer to observe than the effect on T2DM or CV risk. Few reports investigated whether there is a long-term benefit from short-term weight loss as it relates to cancer risk reduction if there is subsequent weight regain to or above baseline. Long-term adherence to changes in lifestyle, including changes in what an individual eats, has been difficult for many obese and overweight individuals, with most regaining all or most of the weight lost 12–24 months after starting the lifestyle changes. Time-restricted eating, which does not require a reduction in what or how much one eats, but rather only when, demonstrates early evidence of good diet adherence [82], though long-term studies are needed to validate this. However, reports lasting ≥1 year are needed to confirm this and assess the effects on weight loss. In conclusion, sustained dietary adherence is the most important factor in long-term improvements in health. Additional reports that address which strategies lead to long-term (≥1 year) diet adherence on weight loss, with or without the use of medication to sustain adherence, are needed. Precision nutrition approaches that assess the optimal diet for a given individual are needed. Diet intervention reports that include medications are needed to address adherence after medication is stopped, medication side effects, and impact on cancer risk. Moreover, there is a need for more RCTs that include men, studies with substantive representation of individuals from diverse ethnic and socioeconomic backgrounds, and studies that address the effects of the same dietary strategy among different ethnic groups, age groups, or health conditions. Finally, the RCTs that we reviewed rarely address hard cancer endpoints or mechanisms driving the associations. Future research should be designed to better determine if the relationships found with cancer risk are mere associations or causally related.

## Figures and Tables

**Table 1:** 51 studies that met our search criteria.

1st Author	Year	Study	Number Subjects		Subjects	Diet Type, Other Interventions ^1,2^		Study Length	1^o^ Outcome	1^o^ or 2^o^ Study	Finding
			Male (M)	Female (F)		ISO vs. LCD	Intervention Nutrient Composition				
Beresford [12]	2006	WHI-DMT	0	48,835	post	ISO	LFD: 20% fat	avg 8.1 y	BC and CRC incidence	1	did not lower CRC incidence
Black [13]	1995	LFD vs. control	66	35	non-melanoma skin cancer (NMSC)	ISO	LFD: 20% fat	2 y	New NMSC	1	fewer new NMSC LFD group in last 8 months
Black [14]	1998	LFD vs. control in AK or skin ca	70	45	skin CA patients	ISO	LFD: 20% fat	2 y	New AK and NMSC	2	LCD group saw fewer AK overall, and fewer new NMSC in last 8 months
Botteri [15]	2018	low chol/low cal/stop smoking	1216	0	hi CAD risk men	LCD	↓ saturated fat, stop smoking	5 y	CV mortality	2	lower cancer risk HR = 0.69 at 25 years, but not beyond
Boyd [16]	1988	dense breasts	0	295	dense breasts	ISO	LFD: 15% fat	1 y	∆ Breast density	1	no change in breast density
Boyd [17]	1997	LFD	0	220	pre with dense breasts	ISO	LFD: 15% fat	2 y	Sex hormone levels	2	lower estradiol, progesterone in LFD group
Brown [18]	2022	LCD/EX/both/control	0	269	BC survivors, BMI > 25 kg/m^2^	LCD	LCD: goal 10% ↓ weight	1 y	Sex hormone levels	1	no ∆ with diet, EX, or both vs. control
Bruno [19]	2021	MedD in BC survival/high metab risk	0	1344	BC survivors	LCD	LCD, low meat protein & fat	1 y	Adherence to MedD with improve metabolic syndrome parameters	1	improvement in metabolic syndrome parameters, weight loss related to MedD adherence
Byrd [20]	2022	PPT	218	150	hx CR adenomas	ISO	LFD: 20% fat	4 y	Adenoma recurrence	2	High baseline bile acids associated with recurrence, no effect of diet
Caan [21]	2009	WHI diet modification trial	0	48,835	post	ISO	LFD: 20% fat	avg 8.1 y	Risk BC based on hot flashes (HF)	2	HF predicted lower risk of BC among Tx group (HR = 0.27)
Campbell [22]	2012	LCD/EX/both/control	0	439	post: BMI > 25 kg/m^2^	LCD	LCD: goal 10% ↓ weight	1 y	intervention in sex hormones	1	wt loss ↓ estrogens and testosterone
Chlebowski [23]	2006	WHI nutrition study	0	2437	BC survivors	ISO	LFD: 20% fat	5 y	BC recurrence	1	may lower recurrence (*p* = 0.077 log rank, *p* = 0.034 Cox model)
Chlebowski [24]	2017	WHI-DMT	0	48,835	post	ISO	LFD: 20% fat	8.5 y median	BC and CRC incidence	2	lower risk of dying from BC HR = 0.65
Chlebowski [25]	2018a	WHI-DMT	0	48,835	post	ISO	LFD: 20% fat	8.5 y median	BC and CRC incidence	2	increased BC survival with LFD after a diagnosis of BC
Chlebowski [26]	2018b	WHI-DMT		48,835	post	ISO	LFD: 20% fat			2	among women who developed BC, lower risk of dying overall (from any cause) after 17.7 yrs median follow-up
Chlebowski [7]	2020	WHI-DMT	0	48,835	post	ISO	LFD: 20% fat	8.5 y median	BC and CRC incidence	2	if developed BC, lower risk of dying overali and from BC after 19.6 y median follow-up
deLorgeril [27]	1998	Lyon Heart Diet	392	203	CV disease	ISO	MedD	4 y	Cardiac mortality	2	↓ total mortality (HR = 0.56), lower cancers(HR = 0.61)
Duggan [28]	2015	LCD + vitD3 vs. LCD + placebo	0	218	post/BMI ≥ 25 kg/m^2^/low vitD	LCD	LCD: goal 10% ↓ weight	1 y	Inflamm biomarkers	1	lower IL-6 with both wt loss ≥ 5% and vitD3 supplementation
Duggan [29]	2016	LCD/EX/both/control	0	439	post/BMI ≥ 25 kg/m^2^	LCD	LCD: goal 10% ↓ weight	1 y	Angiogenesis biomarkers	2	weight loss associated with ↓ PAI-1, PEDF, VEGF
Duggan [30]	2021	LCD/EX/both/control	0	438	post/BMI ≥ 25 kg/m^2^	LCD	LCD: goal 10% ↓ weight	1 y	DII (inflammatory index) and energy-adj DIII (E-DII) biomark	2	LCD and LCD + EX had lower E-DII v. contr, include ghrelin, VEGF, RBC count.
Emond [31]	2011	WHEL study	0	447	post BC suvivor w/o hot flashes	ISO	LFD:15–20% fat	7.3 y mean	BC recurrence	2	lower BC if no HF at baseline due to lowering of testosterone (total and bioavailable)
Flood [32]	2008	PPT if serum available for biomarker analysis	534	216	Recent CR adenoma	ISO	LFD: 20% fat	4 y	Polyp recurrence	2	no effect on IGF-1, insulin, IGFBP-3; lower glucose if lean
Fontana [33]	2016	CALERIE study	218 total; M/F unclear		BMI 22–27.8 kg/m^2^, 20–50 yo	LCD	LCD: goal 25% ↓ weight	2 y	Cardiometabolic effects	2	IGFBP-1 increased, IGF-1, cortisol unchanged
Gamba [34]	2013	WHI-DMT	0	48,835	LFD	ISO	LFD	8.1 y median	BC and CRC incidence	2	skin CA risk: no effect on melanoma or NMSC risk
Gann [35]	2003	LFD, hi fiber	0	213	20–40 yo, healthy	ISO	LFD: 20% fat	1 y	Sex hormone levels	1	nonsignificant lowering of E2
Habermann [36]	2015	NEW study	0	439	post: BMI > 25 kg/m^2^	LCD	LCD: goal 10% ↓ weight	1 y	Sex hormone levels	2	no ∆ in DNA repair capacity
Imayama [37]	2012	NEW study	0	439	post: BMI > 25 kg/m^2^	LCD	LCD: goal 10% ↓ weight	1 y	Inflamm biomarkers	2	LCD and LCD + EX groups lowered IL-6, CRP, serum amyloid, especially if ≥5% wt loss
Jiao [38]	2018	WHI diet mod trial	0	46,200	post	ISO	LFD: 20% fat	8.5 y median	BC and CRC incidence	2	↓ pancreas cancer risk if BMI > 25 kg/m^2^
Lanza [39]	2007	PPT Continued Follow-up Study	529	272	hx CR adenomas	ISO	LFD: 20% fat	8 y	Polyp recurrence	2	no effect on adenoma recurrence
Liu [40]	2022	metformin vs. metformin + ex + diet	72	48	renal cell ca + DM	ISO	custom diet	1 y	Glucose and lipid metabolism, progression-free survival	1	improved glucose and lipid metabolism, better PFS
Martin [41]	2011	LFD	0	4690	hi mammo density	ISO	LFD: 15% fat	10 y mean	Invasive BC	1	no effect on BC risk
Masala [42]	2020	plant based diet/EX/both/control	0	234	post, high breast density	ISO	low saturated fat	2 y	Inflamm biomarkers	1	↓ IL-1α and IL-6 with EX or EX + diet vs. control
Mason [43]	2013	NEW study	0	439	post/BMI ≥ 25 kg/m^2^	LCD	LCD: goal 10% ↓weight	1 y	Intervention in sex hormones	2	no ∆ in IGF-1 or IGFBP-3, but IGF-1/IGFBP-3 ratio ↑ in LCD and LCD + EX group vs. control
McKeown-Eyssen [44]	1994	LFD hi fiber v. control	110	91	history CR polyps	ISO	LFD: 20% fat	2 y	Polyp recurrence	1	no impact on polyp recurrence
Pan [45]	2019	WHI-DMT	0	48,835	post	ISO	LFD: 20% fat	11.4 y mean	BC and CRC incidence	2	lower BC mortality in treatment arm
Pan [46]	2021	WH-DMT	0	48,835	LFD	ISO	LFD: 20% fat	8.5 y median	BC and CRC incidence	2	LFD lowered BC mortality more in participants with 3–4 (vs. fewer) metabolic syndrome components
Peila [47]	2021	WHI-DMT	0	48,835	LFD	ISO	LFD: 20% fat	18.7 y median	BC and CRC incidence	2	no impact on DCIS risk
Pierce [48]	2007	WHEL study		3088	LFD	ISO	LFD: 15–20% fat	6 y	Invasive BC, new or recurrent	1	no impact
Prentice [49]	2006	WHI-DMT	0	48,835	LFD	ISO	LFD: 20% fat	8.1 y mean	BC and CRC incidence	1	non-significant trend to lower BC risk
Prentice [50]	2007	WHI-DMT	0	48,835	LFD	ISO	LFD: 20% fat	8.1 y mean	BC and CRC incidence	2	↓ ovarian cancer risk after ≥4 years on study
Prentice [51]	2019	WHI-DMT	0	48,835	LFD	ISO	LFD: 20% fat	8.5 y median	BC and CRC incidence	1	after 19.6 yr f/u, lower mortality from BC mortality
Rana [52]	2017	3 arm study, all LCD	0	242	post: BMI > 25 kg/m^2^	LCD	LFD, HFD, or walnut rich diet	1 y	Biomarkers of BC	1	IL-6 snp rs1800795 is not related to when IL-6 change occurs
Reeves [53]	2021	LCD	0	159	BC survivors	LCD	LCD: 5–10% ↓weight	1.5 y	Biomarkers of BC	1	improved metabolic syndrome components
Rock [54]	2004	WHEL subgroup study	0	291	BC survivors	ISO	LFD: 15–20% fat	1 y	BC recurrence	2	↓ E2
Rock [55]	2016	LFD/hi fat/walnut rich, hi fat	0	245	BMI > 25 kg/m^2^	LCD	LFD vs. high fat vs. walnut high fat	1 y	Metabolic factors	1	all groups had ↓ CRP and IL-6
Rohan [56]	2008	WHI-DMT	0	48,835	LFD	ISO	LFD: 20% fat	7.7 y mean	BC and CRC incidence	2	benign breast disease not changed
Sansbury [57]	2009	PPT	1229	676	LFD, focus on adherence	ISO	LFD: 20% fat	4 y	Adenoma recurrence	2	↓ adenoma recurrence among dietary “super compliers”
Schatzkin [58]	2000	PPT	1229	676	LFD, hi fiber, vegetables, fruit	ISO	LFD: 20% fat	4 y	Adenoma recurrence	1	LFD did not alter adenoma recurrence
Thomson [59]	2014	WHI-DMT	0	48,835	LFD	ISO	LFD: 20% fat	8.3 y mean	BC and CRC incidence	2	no change in overall BC or CRC risk or mortality, though lower risk of ER+/PR− BC
Toledo [60]	2015	Predimed trial	0	4282	women 60–80 hi CVD risk	ISO	MedD, olive oil/MedD, nuts/control	4.8 y mean	CVD impact	2	↓ (HR = 0.32) BC incidence for MedD c ex virg olive oil, HR = 0.59 vs. control
Vitale [61]	2023	Dedica trial: MedD	0	223	BC survivors	ISO	MedD/MedD + EX + vitamin D	2.7 y	DII impact	1	↑ MedD adherence & ↓ glycemic index each ↓ lowered DII; may impact cancer prognosis & survival

^1^ Many studies included behavioral modification sessions to maximize diet adherence, with the control group continuing their usual diet. % fat indicates fat as a fraction of total calories. ^2^ AK: actinic keratosis; BMI: body mass index; CA: cancer; CR: colorectal; CVD: cardiovascular disease; Dedica: dietary modification, physical activity and vitamin D; DII: dietary inflammatory index: E2: estradiol; ER/PR; estrogen receptor/progesterone receptor; EX: aerobic exercise; ISO: isocaloric; LFD: low fat diet; LCD: low calorie diet; MedD: Mediterranean diet:NEW: Nutrition and Exercise for Women; PFS: progression-free survival; PPT: Polyp Prevention Trial; pre: premenopausal; post: postmenopausal; Pedimed: Prevencion con Dieta Mediterranea; WHEL: Women’s Healthy Eating and Living; WHI-DMT: Women’s Health Initiative Diet Modification Trial; yo: years old.

**Table 2:** Benefits of Isocaloric and Low-Calorie Diets on Cancer and Cancer Biomarkers.

**Primary Reports**					
Isocaloric Studies			Low-Calorie Diet Studies		
Y	N	Total (%)	Y	N	Total (%)
5	8	13 (38)	5	2	7 (71)
**Secondary Reports**					
Isocaloric Studies			Low-Calorie Diet Studies		
Y	N	Total (%)	Y	N	Total (%)
16	6	22 (73)	6	1	6 (86)

**Table 3:** Population Focus of Primary Reports.

**Breast Cancer (BC)**	**Healthy Women ^1^**	**Colorectal Cancer**	
6	9	1	
**Skin Cancer**	**Colorectal Polyps**	**Renal Cell Cancer**	**Total**
1	2	1	20

^1^ Four of the healthy women studies assessed breast density and the impact of diet on density, BC risk, or biomarkers of cancer.

## Supplementary Materials

The following supporting information can be downloaded at: https://www.mdpi.com/article/10.3390/cancers16193296/s1, File S1. Database Search Strategies. Table S1. Risk of Bias Table.
