# Supplementary material for: Long-Term Randomized Controlled Trials of Diet Intervention Reports and Their Impact on Cancer: A Systematic Review
Source: Cancers (Basel). 2024 Sep 27;16(19):3296. doi: 10.3390/cancers16193296 (PMC11475623; doi:10.3390/cancers16193296)
Supplement: Supplementary file 1 [file cancers-16-03296-s001.zip › cancers-3161766-supplementary.pdf]

Supplementary Materials

Supplementary File 1 (File S1). Database Search Strategies

Supplementary Table 1 (Table S1). Risk of Bias Table

Supplementary Table S1 #Assessment of Risk of Bias - NIH Study Assessment Tool

Author Year	Q1	Q2	Q3	Q4	Q5	Q6	Q7	Q8	Q9	Q 10	Q11	Q12	Q13	Q14	Quality Rating
Beresford, 2006	Yes	Yes	No	No	No	Yes	NR	NR	No	Yes	Yes	Yes	Yes	Yes	Good
Black, 1995	Yes	Yes	No	No	No	Yes	Yes	Yes	Yes	No	Yes	Yes	Yes	Yes	Good
Black, 1998	Yes	Yes	No	No	No	Yes	No	No	Yes	Yes	Yes	Yes	Yes	Yes	Good
Botteri, 2018	Yes	NR	No	No	No	Yes	No	NR	NR	No	Yes	CD	Yes	Yes	Good
Boyd, 1988	Yes	Yes	No	No	No	Yes	No	No	Yes	No	Yes	Yes	Yes	Yes	Good
Boyd, 1997	Yes	Yes	No	No	No	Yes	Yes	Yes	Yes	Yes	Yes	No	No	No	Good
Brown, 2022	Yes	Yes	No	No	No	No	CD	NR	Yes	No	Yes	Yes	Yes	Yes	Good
Bruno, 2021	Yes	Yes	No	No	No	Yes	Yes	Yes	No	Yes	Yes	No	Yes	No	Good
Byrd 2022	Yes	Yes	No	No	No	CD	NR	Yes	Yes	No	Yes	No	Yes	No	Good
Caan, 2009	Yes	Yes	No	No	No	Yes	Yes	Yes	No	Yes	Yes	Yes	Yes	Yes	Good
Campbell, 2012	Yes	Yes	No	No	Yes	Yes	Yes	Yes	Yes	Yes	Yes	Yes	Yes	Yes	Good
Chleborski, 2018	Yes	Yes	No	No	No	Yes	Yes	Yes	Yes	Yes	Yes	No	Yes	Yes	Good
Chlebowski, 2006	Yes	Yes	No	No	No	Yes	No	No	Yes	Yes	Yes	Yes	Yes	Yes	Good
Chlebowski, 2017	Yes	Yes	No	No	No	NR	NR	NR	NR	Yes	Yes	No	Yes	Yes	Good
Chlebowski, 2018	Yes	Yes	No	No	No	Yes	Yes	Yes	Yes	Yes	Yes	Yes	Yes	Yes	Good
Chlebowski, 2020	Yes	Yes	No	No	No	Yes	NR	NR	NR	Yes	Yes	NR	Yes	Yes	Good
de Lorgeril, 1998	Yes	Yes	No	No	No	Yes	Yes	No	Yes	Yes	Yes	Yes	Yes	Yes	Good
Duggan, 2015	Yes	Yes	Yes	Yes	Yes	Yes	Yes	Yes	Yes	Yes	Yes	Yes	Yes	Yes	Good
Duggan, 2016	Yes	Yes	No	No	No	Yes	Yes	Yes	Yes	Yes	Yes	Yes	Yes	Yes	Good
Duggan, 2021	Yes	Yes	No	No	No	Yes	Yes	Yes	NR	Yes	Yes	NR	Yes	Yes	Good
Emond, 2011	Yes	Yes	No	No	No	Yes	Yes	NR	Yes	Yes	Yes	Yes	Yes	Yes	Good
Flood, 2008	Yes	Yes	No	No	No	Yes	Yes	Yes	Yes	Yes	Yes	Yes	Yes	Yes	Good
Fontaina, 2016	Yes	Yes	No	No	No	Yes	NR	NR	NR	Yes	Yes	NR	Yes	Yes	Good
Gamba, 2013	Yes	Yes	No	No	No	Yes	Yes	Yes	Yes	Yes	Yes	Yes	Yes	Yes	Good
Gann, 2003	Yes	Yes	No	No	No	Yes	Yes	Yes	Yes	Yes	Yes	Yes	Yes	Yes	Good
Habermann, 2015	Yes	Yes	No	No	No	Yes	NR	NR	Yes	Yes	Yes	Yes	Yes	Yes	Good
Imayama, 2012	Yes	Yes	Yes	Yes	No	Yes	Yes	No	Yes	Yes	Yes	Yes	Yes	Yes	Good

Jiao, 2018	Yes	Yes	No	No	No	Yes	Yes	Yes	Yes	Yes	Yes	Yes	Yes	No	Good
Lanza, 2007	Yes	Yes	No	No	No	Yes	No	Yes	No	Yes	Yes	Yes	Yes	No	Good
Liu, 2022	Yes	Yes	No	No	No	Yes	NR	NR	NR	Yes	Yes	No	Yes	Yes	Good
Martin, 2011	Yes	Yes	No	No	No	Yes	Yes	Yes	Yes	Yes	Yes	Yes	Yes	Yes	Good
Masala, 2020	Yes	Yes	No	No	No	Yes	Yes	Yes	Yes	Yes	Yes	Yes	Yes	Yes	Good
Mason 2013	Yes	Yes	No	No	No	Yes	Yes	Yes	Yes	Yes	Yes	Yes	Yes	Yes	Good
McKeown-Eyssen, 1994	Yes	Yes	No	No	No	Yes	No	Yes	No	Yes	Yes	Yes	Yes	Yes	Good
Pan, 2019	Yes	Yes	No	No	No	Yes	Yes	Yes	Yes	Yes	Yes	Yes	Yes	Yes	Good
Pan, 2021	Yes	Yes	No	No	No	Yes	Yes	Yes	Yes	Yes	Yes	Yes	Yes	Yes	Good
Peila, 2021	Yes	Yes	No	No	No	Yes	Yes	No	NR	Yes	Yes	Yes	Yes	Yes	Good
Pierce, 2007	Yes	Yes	No	No	Yes	Yes	NR	NR	Yes	Yes	Yes	NR	Yes	Yes	Good
Prentice, 2006	Yes	Yes	No	No	No	Yes	Yes	Yes	Yes	Yes	Yes	Yes	Yes	Yes	Good
Prentice, 2007	Yes	Yes	No	No	Yes	Yes	NR	NR	No	Yes	Yes	NR	Yes	Yes	Good
Prentice, 2019	Yes	Yes	No	No	No	Yes	Yes	Yes	Yes	Yes	Yes	Yes	Yes	Yes	Good
Rana, 2017	Yes	Yes	No	No	No	NR	NR	NR	NR	Yes	Yes	Yes	Yes	Yes	Good
Reeves, 2021	Yes	Yes	No	No	No	Yes	Yes	No	NR	Yes	Yes	Yes	Yes	Yes	Good
Rock, 2004	Yes	Yes	No	No	No	Yes	Yes	Yes	Yes	Yes	Yes	Yes	Yes	Yes	Good
Rock, 2016	Yes	Yes	No	No	No	Yes	NR	NR	NR	Yes	Yes	NR	Yes	Yes	Good
Rohan, 2008	Yes	Yes	No	No	No	Yes	Yes	Yes	Yes	Yes	Yes	Yes	Yes	Yes	Good
Sansbury, 2009	Yes	Yes	No	No	No	Yes	Yes	Yes	Yes	Yes	Yes	Yes	Yes	Yes	Good
Schatzkin, 2000	Yes	Yes	No	No	Yes	Yes	Yes	Yes	Yes	Yes	Yes	Yes	Yes	Yes	Good
Thomson, 2014	Yes	Yes	No	No	No	Yes	Yes	Yes	Yes	Yes	Yes	Yes	Yes	Yes	Good
Toledo, 2015	Yes	Yes	No	No	Yes	Yes	NR	NR	NR	Yes	Yes	NR	Yes	Yes	Good
Vitale, 2022	Yes	Yes	No	No	No	Yes	Yes	Yes	Yes	Yes	Yes	Yes	Yes	Yes	Good
*CD (Cannot determine); NR (Not reported)															

NIH Study Quality Assessment Tools
Q1. Was the study described as randomized, a randomized trial, a randomized clinical trial, or an RCT?
Q2. Was the method of randomization adequate (i.e., use of randomly generated assignment)?

Q3. Was the treatment allocation concealed (so that assignments could not be predicted)?
Q4. Were study participants and providers blinded to treatment group assignment?
Q5. Were the people assessing the outcomes blinded to the participants' group assignments?
Q6. Were the groups similar at baseline on important characteristics that could affect outcomes (e.g., demographics, risk factors, co-morbid conditions)?
Q7. Was the overall drop-out rate from the study at endpoint 20% or lower of the number allocated to treatment?
Q8. Was the differential drop-out rate (between treatment groups) at endpoint 15 percentage points or lower?
Q9. Was there high adherence to the intervention protocols for each treatment group?
Q10. Were other interventions avoided or similar in the groups (e.g., similar background treatments)?
Q11. Were outcomes assessed using valid and reliable measures, implemented consistently across all study participants?
Q12. Did the authors report that the sample size was sufficiently large to be able to detect a difference in the main outcome between groups with at least 80% power?
Q13. Were outcomes reported or subgroups analyzed prespecified (i.e., identified before analyses were conducted)?
Q14. Were all randomized participants analyzed in the group to which they were originally assigned, i.e., did they use an intention-to-treat analysis?

Supplementary File S1. Database Search Strategies

Database: PubMed/MEDLINE

Vendor: National Library of Medicine

Date Searched: 3/14/2023

	Concept:	Search Strategy:
#1	Dietary Pattern	"dietary pattern*"[Title/Abstract] OR "diet pattern*"[Title/Abstract] OR "eating pattern*"[Title/Abstract] OR "food pattern*"[Title/Abstract] OR "Diet Therapy"[Mesh] OR "diet therap*"[Title/Abstract] OR "dietary restrict*"[Title/Abstract] OR "Diet, Mediterranean"[Mesh] OR "Mediterranean Diet*"[Title/Abstract] OR "Dietary Approaches To Stop Hypertension"[Mesh] OR "Dietary Approaches To Stop Hypertension Diet*"[Title/Abstract] OR "DASH diet*"[Title/Abstract] OR "Diet, Gluten-Free"[Mesh] OR "Gluten Free diet*"[Title/Abstract] OR "prudent diet*"[Title/Abstract] OR "Diet, Paleolithic"[Mesh] OR "Paleolithic Diet*"[Title/Abstract] OR "Diet, Vegetarian"[Mesh] OR "vegetarian diet*"[Title/Abstract] OR "vegan diet*"[Title/Abstract] OR "Diet, Healthy"[Mesh] OR "healthy diet*"[Title/Abstract] OR "plant based diet*"[Title/Abstract] OR "Diet, Western"[Mesh] OR "western diet*"[Title/Abstract] OR "Nordic Diet*"[Title/Abstract] OR "Okinawan diet*"[Title/Abstract] OR "Diet, Fat-Restricted"[Mesh] OR "Diet, High-Fat"[Mesh] OR "high-fat diet*"[Title/Abstract] OR "low fat diet*"[Title/Abstract] OR "Caloric Restriction"[Mesh] OR "calorie restrict*"[Title/Abstract] OR "caloric restrict*"[Title/Abstract] OR "Diet, Sodium-Restricted"[Mesh] OR "low-sodium diet*"[Title/Abstract] OR "low salt diet*"[Title/Abstract] OR "Fasting"[Mesh] OR "fasting duration"[Title/Abstract:~6] OR "intermittent fasting*"[Title/Abstract]
#2	Cancer Risk	"Neoplasms"[Mesh] OR "neoplasm risk"[Title/Abstract:~4] OR "cancer risk"[Title/Abstract:~4] OR "Cancer Survivors"[Mesh] OR "cancer surviv*"[Title/Abstract] OR "anticancer*"[Title/Abstract] OR "Early Detection of Cancer"[Mesh] OR "early cancer diagnosis"[Title/Abstract:~4]
#3	Adherence	"Guideline Adherence"[Mesh] OR "guideline*"[Title/Abstract] OR "adherence*"[Title/Abstract] OR "Follow-Up Studies"[Mesh] OR " follow-up*"[Title/Abstract] OR "long-term*"[Title/Abstract] OR "longterm*"[Title/Abstract] OR "month*"[Title/Abstract] OR "year*"[Title/Abstract] OR "treatment*"[Title/Abstract] OR "healthy eating ind*"[Title/Abstract] OR "diet index"[Title/Abstract:~2] OR "dietary indic*"[Title/Abstract] OR "diet indic*"[Title/Abstract]
#4	Clinical Trials	"Randomized Controlled Trial" [Publication Type] OR "Randomized Controlled Trials as Topic"[Mesh] OR "Controlled Clinical Trial" [Publication Type:NoExp] OR "RCT"[Title] OR "randomized"[Title/Abstract] OR "Placebos"[Mesh] OR "placebo"[Title/Abstract] OR "drug therapy" [Subheading] OR "randomly"[Title/Abstract] OR "controlled trial"[Title/Abstract] OR "groups"[Title/Abstract] OR "trial"[Title] OR "intervention trial"[Title/Abstract:~10] OR "intervention study"[Title/Abstract:~10]
#4	Limits & Filters	((#1 AND #2 AND #3 AND #4) NOT ("Animals"[Mesh] NOT ("Animals"[Mesh] AND "Humans"[Mesh]))) NOT ("Models, Animal"[Mesh] OR "In Vitro Techniques"[Mesh]) NOT ("news"[Publication Type] OR "letter"[Publication Type] OR "retracted publication"[Publication Type] OR "retraction of publication"[Publication Type] OR "retraction of publication*"[Title/Abstract] OR "retraction notice"[Title] OR "retracted publication"[Title] OR "Congress"[Publication Type] OR "Consensus Development Conference"[Publication Type] OR "conference abstract*"[Title/Abstract] OR

		"conference proceeding*"[Title/Abstract] OR "conference paper*"[Title/Abstract] OR "conference review*"[Title/Abstract]) Filters: English

Database: Embase
Vendor: Elsevier
Date Searched: 3/14/2023

	Concept:	Search Strategy:
#1	Dietary Pattern	'diet therapy'/de OR 'mediterranean diet'/exp OR 'dash diet'/exp OR 'gluten free diet'/exp OR 'paleolithic diet'/exp OR 'vegetarian diet'/exp OR 'healthy diet'/exp OR 'western diet'/exp OR 'low fat diet'/exp OR 'lipid diet'/exp OR 'caloric restriction'/exp OR 'sodium restriction'/exp OR 'fasting'/exp OR 'dietary pattern*':ab,ti OR 'diet pattern*':ab,ti OR 'eating pattern*':ab,ti OR 'food pattern*':ab,ti OR 'diet therap*':ab,ti OR 'dietary restrict*':ab,ti OR 'mediterranean diet**':ab,ti OR 'dietary approaches to stop hypertension diet*':ab,ti OR 'dash diet*':ab,ti OR 'gluten free diet*':ab,ti OR 'prudent diet*':ab,ti OR 'paleolithic diet*':ab,ti OR 'vegetarian diet*':ab,ti OR 'vegan diet*':ab,ti OR 'healthy diet*':ab,ti OR 'plant based diet*':ab,ti OR 'western diet*':ab,ti OR 'nordic diet*':ab,ti OR 'okinawan diet*':ab,ti OR 'high-fat diet*':ab,ti OR 'low fat diet*':ab,ti OR 'calorie restrict*':ab,ti OR 'caloric restrict*':ab,ti OR 'low-sodium diet*':ab,ti OR 'low salt diet*':ab,ti OR 'intermittent fasting*':ab,ti OR ((fasting NEAR/6 duration):ab,ti)
#2	Cancer	'neoplasm'/exp OR 'cancer patient'/exp OR 'early cancer diagnosis'/exp OR 'cancer surviv*':ab,ti OR 'anticancer*':ab,ti OR (('neoplasm*' NEAR/4 'risk*'):ab,ti) OR (('cancer*' NEAR/4 'risk*'):ab,ti) OR (('early cancer*' NEAR/4 'diagnosis*'):ab,ti)
#3	Adherence	'protocol compliance'/exp OR 'follow up'/exp/mj OR 'guideline*':ab,ti OR 'adherence*':ab,ti OR 'follow-up*':ab,ti OR 'long-term*':ab,ti OR 'longterm*':ab,ti OR 'month*':ab,ti OR 'year*':ab,ti OR 'treatment*':ab,ti OR 'healthy eating ind*':ab,ti OR (('diet' NEAR/2 'index'):ab,ti) OR 'dietary indic*':ab,ti OR 'diet indic*':ab,ti
#4	Clinical Trials	'randomized controlled trial'/exp OR 'randomized controlled trial (topic)'/exp OR [randomized controlled trial]/lim OR 'controlled clinical trial'/de OR 'controlled clinical trial (topic)'/de OR 'placebo'/de OR 'drug therapy'/de OR rct:ti OR trial:ti OR 'randomized':ab,ti OR 'placebo':ab,ti OR 'randomly':ab,ti OR 'controlled trial':ab,ti OR 'groups':ab,ti OR (('intervention' NEAR/10 'trial'):ab,ti) OR (('intervention' NEAR/10 'study'):ab,ti)
#4	Limits & Filters	#1 AND #2 AND #3 AND #4 NOT ([animals]/lim NOT ([animals]/lim AND [humans]/lim)) NOT ('animal model'/exp OR 'in vitro study'/exp) NOT ([conference abstract]/lim OR [conference paper]/lim OR [conference review]/lim OR [editorial]/lim OR [letter]/lim OR [note]/lim OR 'retraction of publication':ab,ti OR 'retraction notice':ti OR 'retracted publication':ab,ti) AND ([article]/lim OR [article in press]/lim) AND [humans]/lim

Database: Web of Science (Core Collection)
Vendor: Clarivate Analytics
Date Searched: 3/14/2023

	Concept:	Search Strategy:
#1	Dietary Pattern	TS=("dietary pattern*" OR "diet pattern*" OR "eating pattern*" OR "food pattern*" OR "diet therap*" OR "dietary restrict*" OR "Mediterranean Diet*"[Title/Abstract] OR "Dietary Approaches To Stop Hypertension Diet*" OR "DASH diet*" OR "Gluten Free diet*" OR "prudent diet*" OR "Paleolithic Diet*" OR "vegetarian diet*" OR "vegan diet*" OR "healthy diet*" OR "plant based diet*" OR "western diet*" OR "Nordic Diet*" OR "Okinawan diet*"

		OR "high-fat diet*" OR "low fat diet*" OR "calorie restrict*" OR "caloric restrict*" OR "low-sodium diet*" OR "low salt diet*" OR "intermittent fasting*" OR ("fasting” NEAR/6 “duration")
#2	Cancer Risk	TS=(cancer OR ("neoplasm” NEAR/4 “risk") OR ("cancer” NEAR/4 “risk") OR "cancer surviv*" OR "anticancer*" OR ("early cancer” NEAR/4 “diagnosis"))
#3	Adherence	TS=("guideline*" OR "adherence*" OR " follow-up*" OR "long-term*" OR "longterm*" OR "month*" OR "year*" OR "treatment*" OR "healthy eating ind*" OR ("diet” NEAR/2 “index") OR "dietary indic*" OR "diet indic*")
#4	Clinical Trials	TS=("randomized" OR "placebo" OR "drug therapy" OR "randomly" OR "controlled trial" OR "groups" OR "RCT"[Title] OR "trial"[Title] OR (“intervention” NEAR/10 “trial") OR (“intervention” NEAR/10 “study"))
#4	Limits & Filters	((#4 AND #3 AND #2 AND #1) NOT TS=("animal model*" OR "in vitro*" OR "in vivo*")) NOT TS=("news" OR "letter" OR "retraction of publication*" OR "conference abstract*" OR "conference proceeding*" OR "conference paper*" OR "conference review*") and Proceeding Paper or Editorial Material or Book Chapters or Note or Meeting Abstract (Exclude – Document Types) and English (Languages)
